# Transcriptome and proteome analyses reveal the regulatory networks and metabolite biosynthesis pathways during the development of *Tolypocladium guangdongense*

**DOI:** 10.1016/j.csbj.2020.07.014

**Published:** 2020-07-25

**Authors:** Gangzheng Wang, Min Li, Chenghua Zhang, Huijiao Cheng, Yu Gao, Wangqiu Deng, Taihui Li

**Affiliations:** a State Key Laboratory of Applied Microbiology Southern China, Guangdong Provincial Key Laboratory of Microbial Culture Collection and Application, Guangdong Open Laboratory of Applied Microbiology, Guangdong Institute of Microbiology, Guangdong Academy of Sciences, Guangzhou 510070, China; b College of Agriculture and Animal Husbandry, Tibet University, Nyingchi, 860000 Tibet, China; c South China Agricultural University, Guangzhou 510642, China; d College of Bioscience and Biotechnology, Hunan Agricultural University, Changsha 410128, China

**Keywords:** *Cordyceps*, Omics analysis, Correlation analysis, Bioactive ingredient biosynthesis-related enzymes, growth and development

## Abstract

*Tolypocladium guangdongense* has a similar metabolite profile to *Ophiocordyceps sinensis*, a highly regarded fungus used for traditional Chinese medicine with high nutritional and medicinal value. Although the genome sequence of *T. guangdongense* has been reported, relatively little is known about the regulatory networks for fruiting body development and about the metabolite biosynthesis pathways. In order to address this, an analysis of transcriptome and proteome at differential developmental stages of *T. guangdongense* was performed. In total, 9076 genes were found to be expressed and 2040 proteins were identified. There were a large number of genes that were significantly differentially expressed between the mycelial stage and the stages. Interestingly, the correlation between the transcriptomic and proteomic data was low, suggesting the importance of the post-transcriptional processes in the growth and development of *T. guangdongense*. Among the genes/proteins that were both differentially expressed during the developmental process, there were numerous heat shock proteins and transcription factors. In addition, there were numerous proteins involved in terpenoid, ergosterol, adenosine and polysaccharide biosynthesis that also showed significant downregulation in their expression levels during the developmental process. Furthermore, both tryptophan and tryptamine were present at higher levels in the primordium stage. However, indole-3-acetic acid (IAA) levels continuously decreased as development proceeded, and the enzymes involved in IAA biosynthesis were also clearly differentially downregulated. These data could be meaningful in studying the molecular mechanisms of fungal development, and for the industrial and medicinal application of macro-fungi.

## Graphical abstract

Workflows of transcriptome and proteome analyses across the differentially developmental stages of *C. guangdongensis*. My, Mycelial stages; P, Primordial stage; YFB, Young fruiting body stage; DFB, Developed fruiting body stage; MFB, Mature fruiting body stage.

**Figure:**
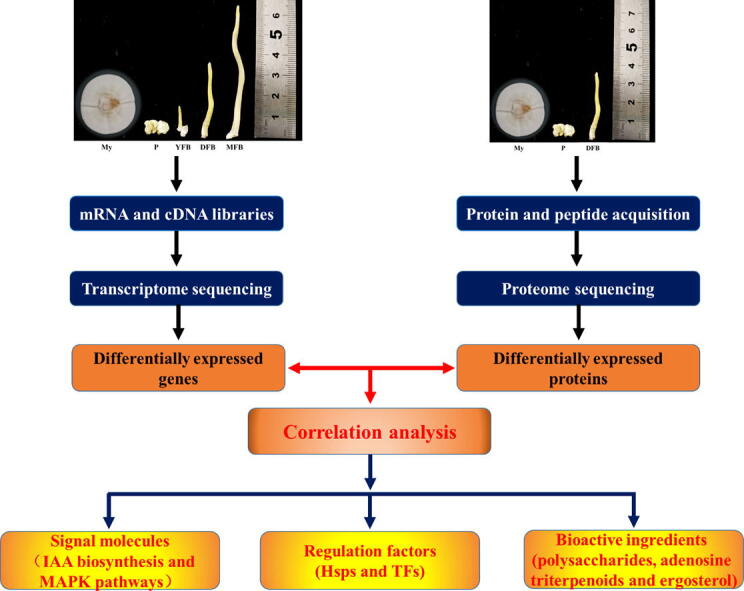

## 1 Introduction

In China, the fruiting bodies of some *Cordyceps* s.l. spp. are well-known traditional medicines because of their anticancer, immunomodulation, and anti-impotence effects [1], [2], [3]. Up to now, only four species of *Cordyceps* s.l., namely *C. militaris*, *C. cicadae*, *Ophiocordyceps sinensis*, and *Tolypocladium guangdongense* previously named as *C. guangdongensis*, have been approved by the Chinese government as medicines or edible fungi, although hundreds of species have been found. *T. guangdongense* grows on the hypogeous tubers of *Elaphomyces* fungi, and is different from other *Cordyceps* s.l. species, which grow on insects [4]. To date, all four of these species have been successfully cultivated, and *C. militaris* has the largest cultivated production, and the rest have only relatively very low cultivated yields [5], [6], [7], [8], [9].

The fruiting body of *T. guangdongense*, which was approved as a novel food source by the Chinese government in 2013, contains numerous active and nutritional compounds such as high levels of cordycepic acid, adenosine, polysaccharides, micro-elements, and vitamins [10], [11]. Based on animal tests, it has been shown that its fruiting body had a clear positive effect in improving the symptoms of several diseases, such as infection with the influenza virus H9N2 and chronic renal failure [12], [13]. In addition, based on the metabolomics analysis, *T. guangdongense* has many similar metabolites to *O. sinensis* (unpublished work), but can be more easily cultivated. These data suggest that *T. guangdongense* could have a range of application in the functional food and medicine industries, and is worthy of further study from many aspects.

In one study, it was found that the mycelia of *T. guangdongense* grew very slowly in PDA medium, achieving a diameter of 2.72 cm after 14 days of growth. The formation and growth of the primordia were easily affected by temperature, light and other factors [5]. Therefore, exploring the molecular mechanism of primordial formation and fruiting body development is very important for improving the quality and yield of *T. guangdongense* and accelerating its industrialization. Recently, the genome of *T. guangdongense* and the gene expression profiles under different light illumination times have been reported by our team [14], [15]. Nevertheless, we do not yet have a comprehensive understanding of its growth and development. The regulatory network and the function of the genes are also unknown.

Advances in omics technologies such as transcriptomics and proteomics have clearly facilitated the comprehensive analysis of gene and protein expression levels under multiple experimental conditions. For example, gene and protein expression profiling have provided a viewpoint into the genes and proteins involved in nutrition transport in *Morchella importuna* [16], bioactive metabolites in *Hericium erinaceus* [17], temperature stress in *Lentinula edodes* [18], Cd^2+^ stress in *Pleurotus eryngii* [19], special odor formation in *Schizophyllum commune* [20] and fruiting body development in *Flammulina velutipes* and *Dictyophora indusiata* [21], [22].

In the cases of the *Cordyceps* s.l. species, there have been transcriptomic and proteomic studies performed in *O. sinensis*, *C. militaris* and *C. cicadae*. Transcriptome analyses in *O. sinensis* have identified genes in the key pathways involved in fruiting body and sexual development-related genes at differential developmental stages [23], [24]. It has been proven that the levels of nutrients between artificially cultured and wild *O. sinensis* samples were virtually the same in terms of nucleosides, nucleotides and adenosine from the perspective of the proteome and metabolome [25]. Using multi-omics studies in *C. militaris*, researchers have uncovered gene expression differences between the mycelium and the fruiting body [26], the transcriptional regulation of central carbon metabolism on sucrose or glucose medium [27], and genes related to the biosynthesis of carotenoids [28]. In *C. cicadae* and *C. kyushuensis*, omics data have revealed the nature of asexual-fruiting and the genes related to cordycepin and pentostatin biosynthesis [29], [30]. These findings show that omics technologies can be effectively used for the analysis of molecular mechanisms in *Cordyceps* fungi.

The expression patterns of genes and proteins are likely a reflection of dynamic changes in gene and protein expression during the growth and development of *T. guangdongense*. Nevertheless, the transcriptome or proteome of *T. guangdongense* have not been described. Therefore, in the present study, fungal samples from differential developmental stages were collected from *T. guangdongense*, and used to perform transcriptome and proteome analyses. Hub genes were detected using weighted gene co-expression network analysis (WGCNA). It is hoped that our viewpoint of the potential molecular mechanisms involved in the developmental process could be substantially improved with the help of comprehensive analyses of the transcriptome and proteome. These data should increase the knowledge of the complex mechanisms regulating primordial formation and fruiting body development, and provide theoretical support for further improvements in cultivation techniques for *T. guangdongense* and for its application in the health-care industry.

## 2 Materials and methods

### 2.1 Sample collection at differential developmental stages

*T. guangdongense* strain CCTCCM206051 was used as the test strain, and was deposited in the China Center for Type Culture Collection. *T. guangdongense* was cultivated as described in a previous study [5]. After growth for 20 d on PDA medium at 23℃, mycelia from *T. guangdongense* were transferred into rice medium and incubated for 6 days in the dark at 23℃ and 60–70% relative humidity. For primordium formation and fruiting body growth, the cultivation room was ventilated daily for 30 min and illuminated (500 lx) for 10 h. Using a previously described method [30], samples of the mycelium (My), primordium (P), young fruiting body (YFB) and mature fruiting body (MFB) were collected. In addition, a sample of the stroma with length 2.5–4 cm was designated as the developed fruiting body (DFB). Three replicate samples were prepared for each developmental stage. After collection, all samples representing the five differentially developmental stages were immediately frozen in liquid nitrogen and then stored at −80 °C prior to RNA and protein isolation.

### 2.2 RNA isolation and sequencing

Total RNA was extracted from all samples using a TRIzol Kit (Invitrogen, Dalian, China) according to the manufacturer’s instruction. After qualitative and quantitative assessment of the RNA samples using an Agilent 2100 Bioanalyzer (Agilent Technologies, Palo Alto, CA, USA), the mRNAs were enriched using oligo-dT magnetic beads, and then fragmented and transcribed into cDNA using random oligonucleotides. The five cDNA libraries were sequenced on a BGISEQ-500 platform at Beijing Genomics Institute (BGI)-Shenzhen, Wuhan, China.

### 2.3 Transcriptome data processing

After removing reads containing poly-N, sequencing adapters and low-quality reads from the raw data, the clean reads were mapped to the *T. guangdongense* genome using HISAT2 and Bowtie2 [31], [32]. The RSEM package was used for calculating the expression levels of genes, and the differential expression analysis was performed using DESeq2 with a Q value < 0.05 [33], [34]. Genes with a false discovery rate (FDR) of ≤ 0.001 and an absolute value of log_2_Ratio ≥ 1 were designated as differentially expressed genes (DEGs). The analysis of gene ontology (GO) and Kyoto Encyclopedia of Genes and Genomes (KEEG) pathway enrichment was performed using Phyper based on the Hypergeometric test [35]. In addition, a weighted correlation network analysis (WGCNA) was performed to analyze the gene co-expression network and identify hub genes [36].

### 2.4 Protein extraction

Mycelia, primordia and developmental fruiting bodies (0.1 g) from each group (three replicates for each group) were homogenized in 0.5 mL of extraction buffer (20 mM Tris-HCl pH = 7.4, 100 mM EDTA, 2% β-mercaptoethanol, 1 mM DTT and 1% Triton X-100). Following this, 1 mL of saturated phenolic Tresturturated Phenol was added, and the mixture was shaken well for 15 min, followed by centrifugation at 25000 × *g* for 15 min at 4 °C to obtain the supernatant (phenol phase). Then, 5 volumes of pre-cooled 0.1 M ammonium acetate in methanol supplemented with 10 mM dithiothreitol (DTT) was added to precipitate the proteins at −20 °C for 2 h. The pelleted proteins were washed two times with pre-cooled acetone. After air-drying of the precipitate, a 1 × Cocktail (Sigma, St Louis, MO, USA) was added and the samples was incubated in an ice bath for 5 min, and then centrifuged at 25000 × g for 15 min at 4 °C. Subsequently, 10 mM DTT was added to the precipitate which was then incubated in a water bath at 56 °C for 1 h. The sample was then incubated with 55 mM iodoacetamide for 45 min in the dark. The supernatant containing proteins was quantified using the Bradford method and SDS-PAGE was used to assess the quality of the protein extract [18].

### 2.5 Proteomic data processing

The final protein solution was diluted with 0.5 M TEAB and then digested with trypsin (Promega, Madison, WI, USA) (enzyme: protein ratio of 1:20) at 37 °C for 2 h. Peptides were labelled using the IBT method as previously described [37]. A mass spectrometric analysis of soluble intracellular proteins was undertaken, as previously described [38]. Raw MS/MS data was queried against the *T. guangdongense* genome using the Mascot search engine MaxQuant (version 2.3.02), and was deposited in the ProteomeXchange Consortium (identifier number PXD020391). The peptides labeled with IBTs were quantitatively analyzed using the IQuant software [38]. Proteins with a P < 0.05 and a fold change > 1.50 or < 0.67 were defined as differentially expressed proteins (DEPs). These proteins were classified and grouped using the GO and KEGG databases, and a hypergeometric test was used to analyze the GO and KEGG pathway annotations. The interaction network and subcellular location were predicted using the STRING protein interaction database and WoLF PSORT software [39], [40].

### 2.6 Quantification of tryptophan and IAA

Samples (0.5 g) of mycelia, primordia and fruiting bodies were ground and then shaken for 15 min in 1 mL of buffer containing 50% methanol and 0.1% formic acid. The mixture was subjected to ultrasound for 30 min in an ice bath to ensure complete homogenization of the sample. The samples were kept at 4℃ for 30 min, and centrifuged at 10000 × *g* for 20 min, and the supernatant was collected. The samples were filtered using a 0.3 μm filter plate and were then analyzed by LC-MS/MS (Waters Iclass-AB Sciex 6500) at the Beijing Genomics Institute (BGI)-Shenzhen, Wuhan, China. MultiQuant was used for chromatographic peak extraction and quantitative analysis of tryptophan, tryptamine, and IAA. Standards of these three metabolites were purchased from Yuanye (Shanghai, China).

### 2.7 Quantitative real time polymerase chain reaction (qRT-PCR) verification of gene expression

The mRNA prepared for transcriptome sequencing was also used for qRT-PCR analysis. Sixteen DEGs were selected to verify the reliability of the transcriptome. The gene-specific primers used for gene quantification were designed using Primer Primer5.0. All primers are listed in Table S1. Histone H4 was used as the internal control [41]. The qRT-PCR reaction was performed using Applied Biosystems ABI 7500 (ABI, Foster City, CA, USA) with three biological and technical repeats. The PCR reaction was performed with an initial denaturation for 2 min at 95 °C; 40 cycles of 20 s at 95 °C and annealing at 60 °C for 20 s. The relative expression levels of target genes were calculated using the 2−^△△Ct^ (Ct, cycle threshold) method as described by Livak [42].

## 3 Results

### 3.1 Transcriptome sequencing analysis

**Fig. 1:**
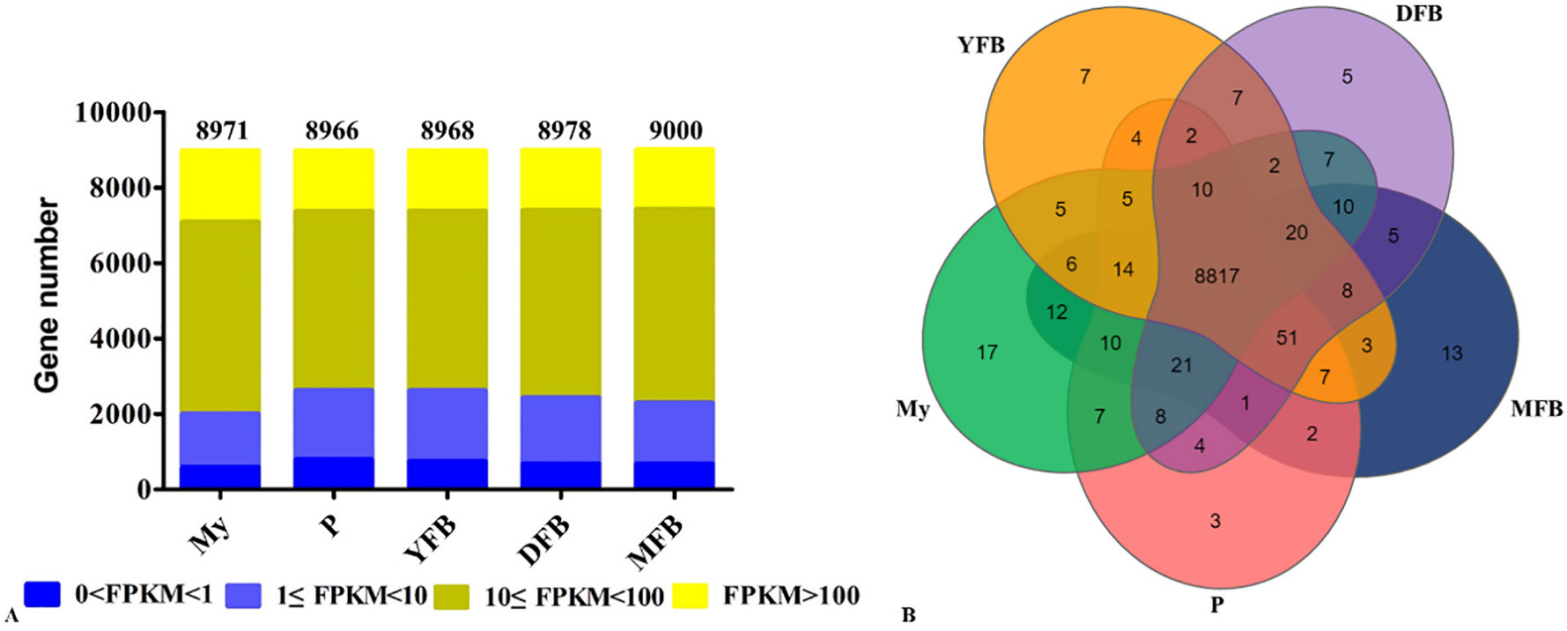
Genes detected and expressed in all samples during the five developmental stages. A, Distribution diagram showing genes with different FPKM values across the five differential developmental stages. B, Venn diagram showing the overlap between genes expressed at each developmental stage. My, Mycelial stage; P, Primordial stage; YFB, Young fruiting body stage; DFB, Developmental fruiting body stage; MFB, Mature fruiting body stage.

To investigate the gene expression changes in *T. guangdongense* during the developmental process, triplicate samples from the five developmental stages were used to construct a total of 15 cDNA libraries after which transcriptome sequencing was performed using BGISEQ-500 sequencing, yielding a total of 334.3 million raw reads. After removing reads containing adapter sequences, poly-N, and the low-quality reads, 330.29 million clean reads were obtained, with a mean of 22.02 million clean reads for each replicate (Table S2). Approximately 80% of the reads from the mycelia could be mapped to the *T. guangdongense* genome, whereas the mapping ratios of the reads from the other samples were>97.46% (Table S2). The Q30 percentages for all the clean reads in the 15 libraries were>93%. Gene expression levels between replicates for each sample exhibited a high Pearson’s correlation coefficient value (Fig. S1), indicating good repeatability between replicates.(See Fig. 1)

more than 99% of genes with FPKM values (>0) were detected in the 15 samples (Fig. 1A and Table S3). Approximately 86.31%, 98%, 98%, 98.13% and 98.37% of the genes were expressed in the My, P, YFB, DFB and MFB stages, respectively (Fig. 1A). A total of 8817 genes were expressed across the five differential developmental stages (Fig. 1B). Based on the gene FPKM values (Table S3), the expression levels of all genes were classified into four categories. During the developmental process, the majority of genes with FPKM values (10 ≤ FPKM 〈1 0 0) were considered to be moderately expressed. Approximately 17% of genes with FPKM value > 100 were considered to be highly expressed, and approximately 25% of genes with FPKM values (0 < FPKM < 10) were considered to have low levels of expression. The number of non-expressed genes (FPKM = 0 or undetected) accounted for around 2% of the total genes at each developmental stage.

**Fig. 2:**
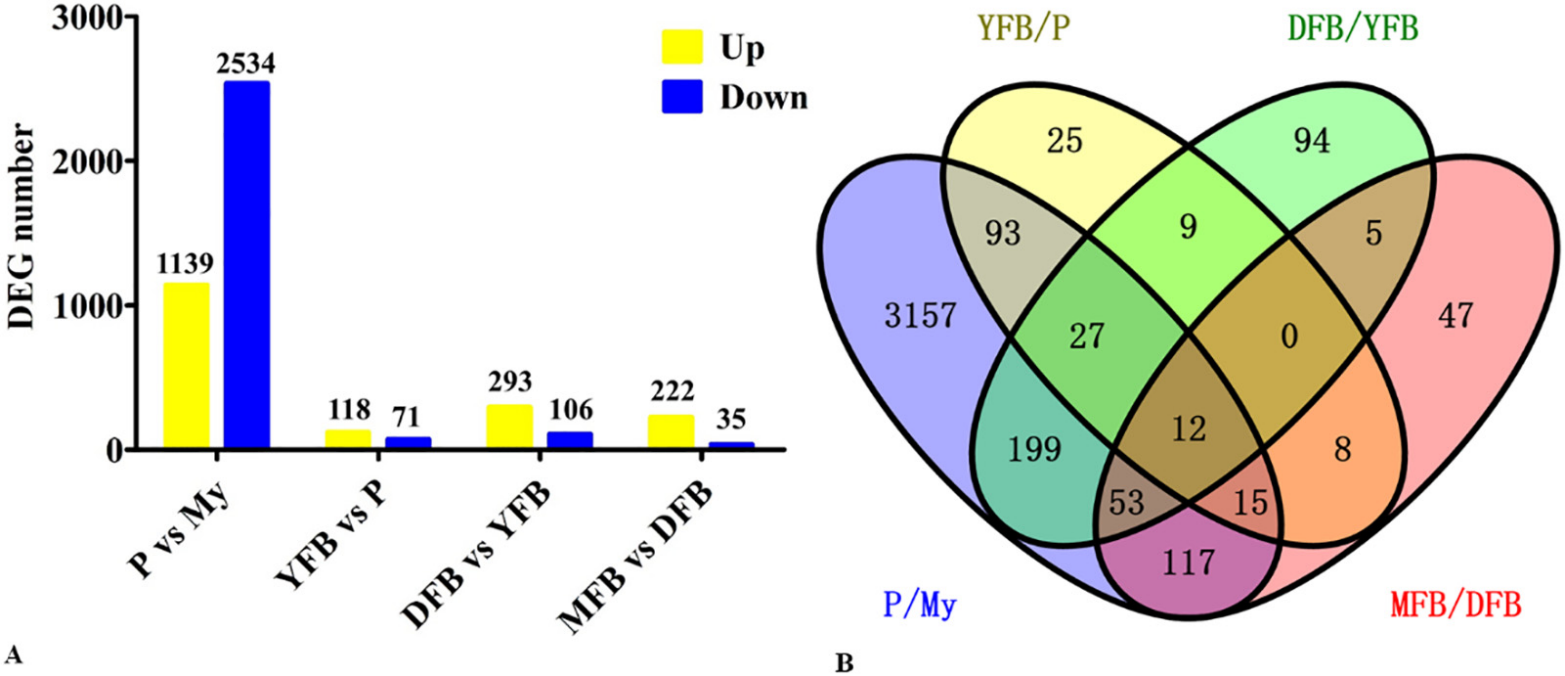
Number of DEGs found between adjacent developmental stages. A, Number of DEGs found between two adjacent development stages. The number of DEGs is shown on the top of the histograms. B, Venn diagram showing the overlaps between the total number of DEGs identified in each of the four comparisons of adjacent development stages. My, Mycelial stage; P, Primordial stage; YFB, Young fruiting body stage; DFB, Developmental fruiting body stage; MFB, Mature fruiting body stage; vs, Versus.

### 3.2 Identification and functional classification of differentially expressed genes across the five developmental stages

Using a principal component analysis (Fig. S2), it was noted that the samples from the five developmental stages, each with three biological replicates, could be clearly divided into two groups, with the mycelial stage separately grouped into one cluster. As shown in Fig. 2A and Table S4, the number of DEGs between the My and P stages was the largest (3673), accounting for 40.14% of the *T. guangdongense* genes, followed by the DFB vs. YFB stages (4 0 0). Fewer DEGs were found in comparisons between the MFB and DFB stages (2 5 7) and between the YFB and P stages (1 8 9). The greatest number of unique DEGs (3157) was found between the MY and P stages, whereas only 25 unique DEGs were found between the YFB and P stages (Fig. 2B). In addition, only 12 DEGs were shared among the four comparisons between adjacent development stages (Fig. 2B). These results suggest that the gene expression profile at the mycelial stage is unique compared to the other developmental stages, whereas the gene expression profiles across the four other developmental stages are very similar to one another with only minor differences being found.

**Fig. 3:**
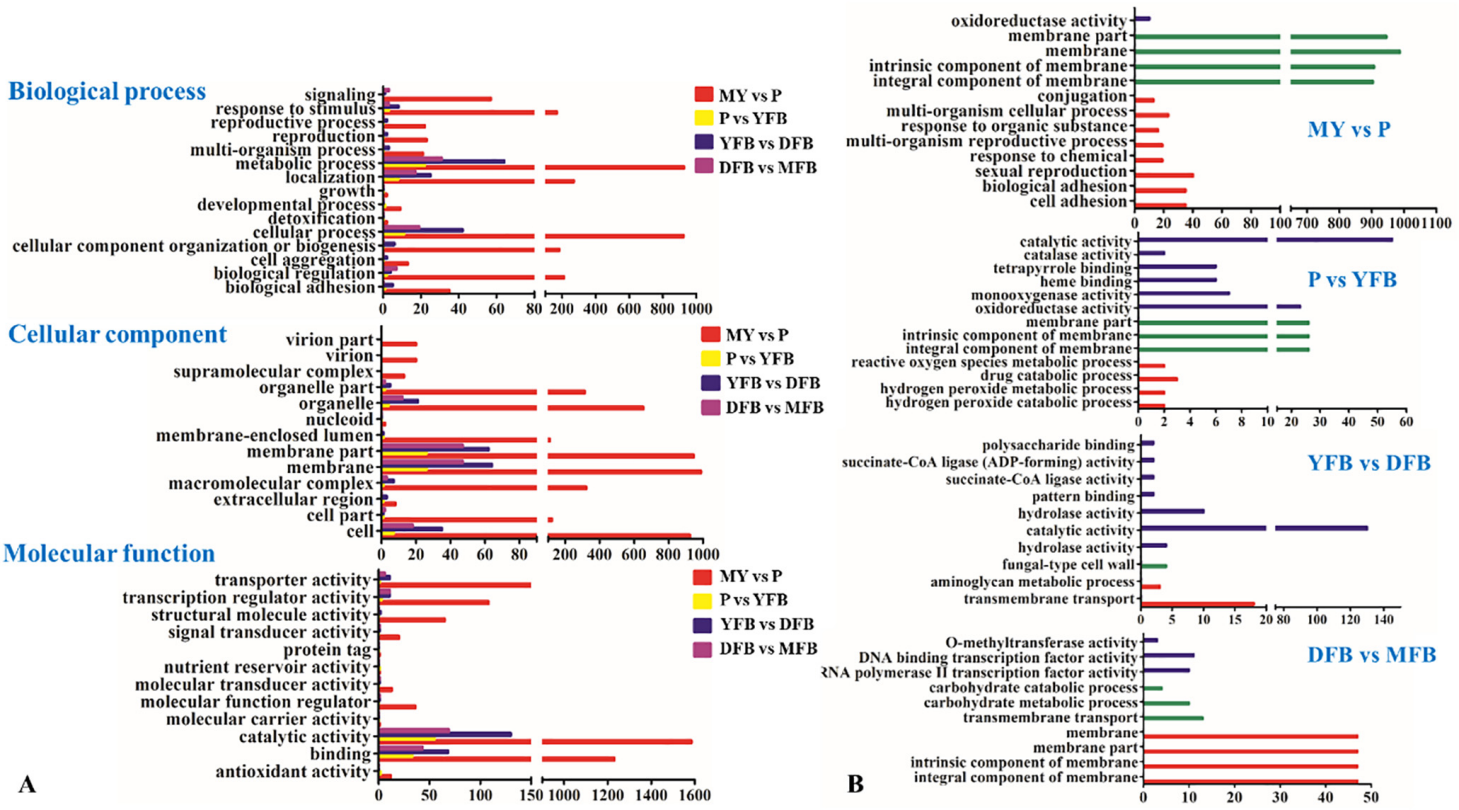
GO functional classification of DEGs in each of the four comparisons of two adjacent development stages. A, Gene ontology term assignments for the DEGs. B, The most enriched GO terms. The red lines represent biological process. The green lines represent cellular component. The blue lines represent molecular function. Only the most significant GO terms with P values < 0.005 were shown. My, Mycelial stage; P, Primordial stage; YFB, Young fruiting body stage; DFB, Developmental fruiting body stage; MFB, Mature fruiting body stage; vs, Versus. (For interpretation of the references to colour in this figure legend, the reader is referred to the web version of this article.)

Classification of DEGs using GO annotation was performed to evaluate the potential functions of these DEGs across the five developmental stages. As shown in Fig. 3A and Table S5, for biological processed (BP) a total of 15 categories were found, including metabolic process (1043), cellular process (9 9 4), localization (3 1 9), biological regulation (2 2 4), cellular component organization or biogenesis, (1 8 8) and response to stimulus (1 8 3). For cellular component (CC), the largest of the 13 categories was related to membrane (1124). Other categories were related to cell (9 8 2), organelle (6 9 0) and macromolecular complex (3 3 2). For molecular function (MF), catalytic activity (1838) and binding activity (1374) were the largest categories. In comparisons between the My and P stages and between the DFB and MFB stages, the most significantly enriched GO assignment was a CC related to membrane (Fig. 3B and Table S5). The most significantly enriched GO terms for the P vs. YFB stages and the YFB vs. DFB stages were in the MF category involved in catalytic activity. In addition, oxidoreductase and monooxygenase activities were found in a comparison of the P vs. YFB stages.

A KEGG pathway analysis was performed to further assess the functions of the DEGs across the five developmental stages (Table S6). In all four of the comparisons of two adjacent development stages, the two pathways with the greatest number of DEGs were related to MAPK signaling and amino sugar and nucleotide sugar metabolism. This was followed by endocytosis and RNA transport in three of the four comparisons (My vs P, YFB vs DFB and DFB vs MFB), whereas biosynthesis of antibiotics and mitophagy were found in the comparison of the P vs. YFB stages. The KEGG pathway enrichment also showed that the diterpenoid pathway was enriched in the comparison of P vs. YFB (P value of 0.002287), and the endocytosis pathway was enriched in the comparison of the YFB vs. DFB stages (P value of 0.0008063).

### 3.3 Gene co-expression network analysis

**Fig. 4:**
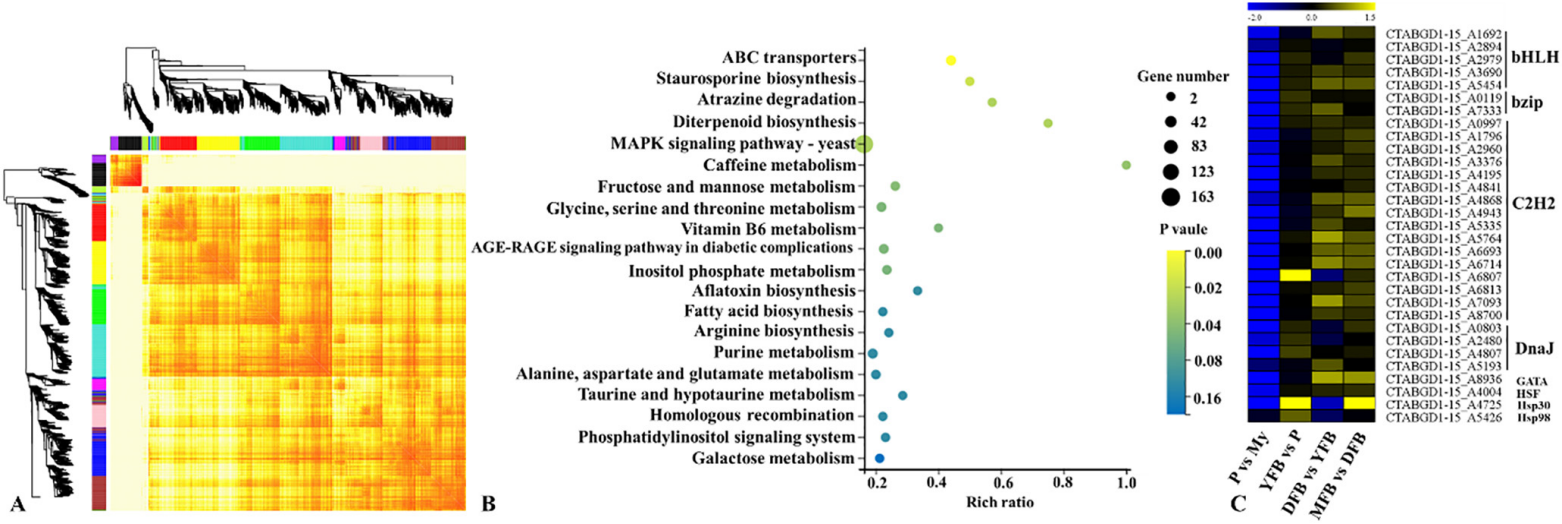
KEGG pathway and expression analyses of hub genes obtained using WGCNA. A, Hierarchical cluster tree showing the 26 co-expression modules labeled with different colors. Every leaf represents one gene. B, KEGG pathway enrichment analysis of 1386 hub genes identified by WGCNA. Pathways with P values < 0.05 were significantly enriched. C, Differential expression levels of the indicated transcription factors and heat shock protein genes obtained from hub genes in each of the four comparisons of two adjacent stages. My, Mycelial stage; P, Primordial stage; YFB, Young fruiting body stage; DFB, Developmental fruiting body stage; MFB, Mature fruiting body stage; vs, Versus.

A WGCNA analysis was performed to gain a comprehensive view of the gene expression patterns during the growth and development process. A total of 1386 hub genes identified from the transcriptome data were divided into 26 modules based on the similarity in their expression patterns (Fig. 4A). As shown in Fig. 4B, the significantly enriched pathways involved ABC transporters (membrane transport), staurosporine biosynthesis (biosynthesis of other secondary metabolites), atrazine degradation (xenobiotics biodegradation and metabolism), diterpenoid biosynthesis (metabolism of terpenoids and polyketides) and MAPK signaling pathway-yeast (signal transduction). After removing genes with FPKM values < 10 in all the five stages, 1135 genes were obtained (Table S7). Among these 12 genes were differentially expressed in the four comparisons of two adjacent stages, namely Hsp30, cytochrome P450, *exo*-beta-1,3-glucanase and nine hypothetical proteins. A total of 961 genes showed higher expression levels at the mycelial stage, whereas only 29 genes were upregulated at the primordial stage compared to the mycelial stage. MAPK signaling pathway-yeast and amino sugar and nucleotide sugar metabolism pathway were significantly enriched pathways at the P vs. My stages (Fig. S3). The downregulated genes included three DnaJ genes, Hsp30 and 25 TFs (transcription factors), including 16 C2H2, 5 bHLH, 2 bZIP, 1 GATA and 1 HSF class TFs (Fig. 4C). In the comparison between the YFB and P stages, 31 and 30 DEGs, including one C2H2 transcription factor and Hsp30, were downregulated and upregulated respectively at the YFB stage. In a comparison of the YFB vs. P stages, diterpenoid biosynthesis and fatty acid degradation pathways were obviously enriched, and the MAPK signaling pathway-yeast had the most DEGs (Fig. S3). A total of 89 DEGs (30 downregulated and 59 upregulated) were identified in the comparison of the DFB vs. YFB stages with the endocytosis pathway being significantly enriched (Fig. S3). During the process of fruiting body maturation (MFB vs. DFB), 62 genes were clearly upregulated in the mature fruiting body, and two pathways involved in aflatoxin biosynthesis and staurosporine biosynthesis were also enriched (Fig. S3).

### 3.4 Proteomics overview

**Fig. 5:**
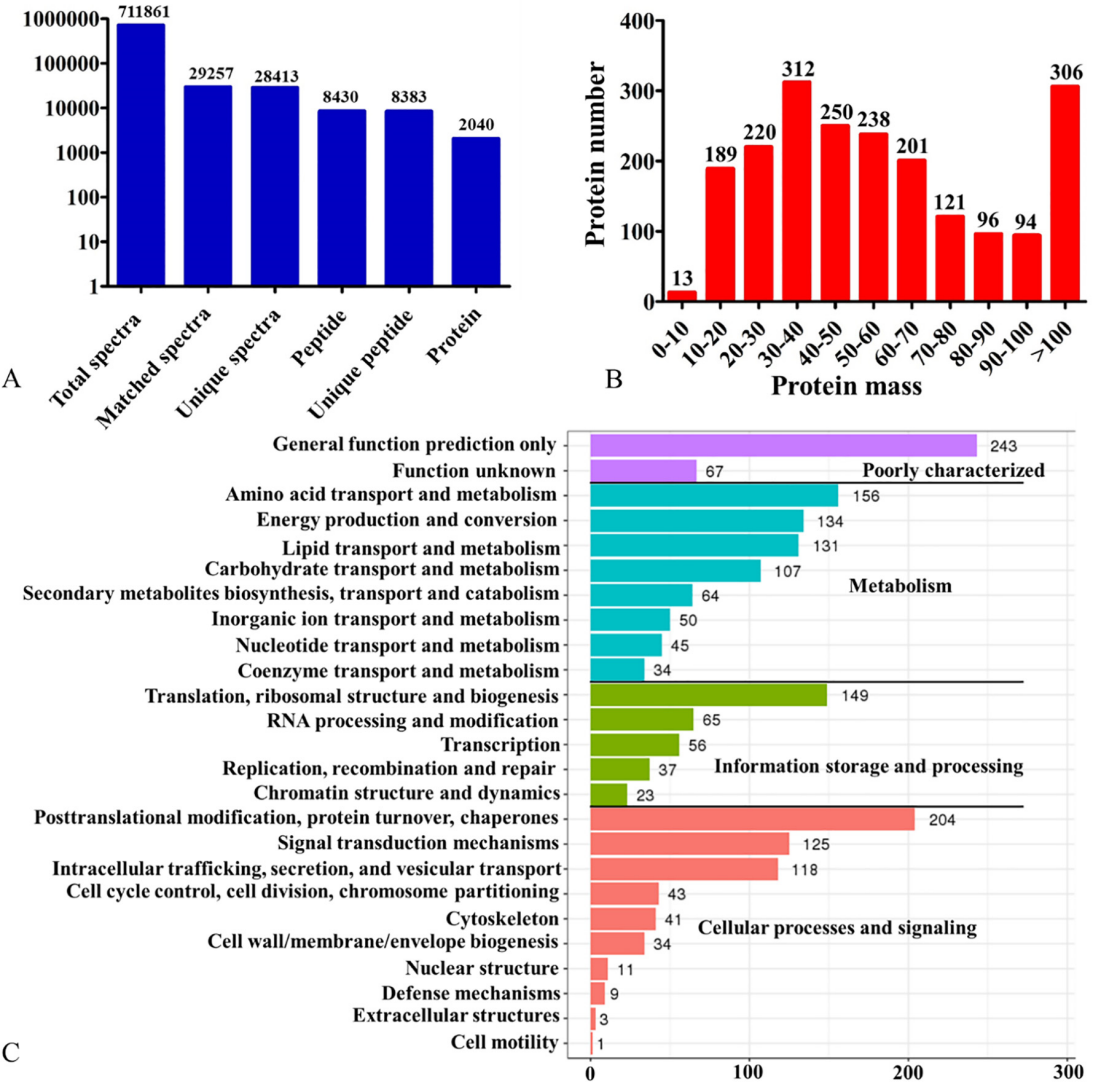
Overview of the *T. guangdongense* proteome. A, The numbers of tandem mass spectrometry spectra and the number of proteins and unique peptides identified by the proteome analysis. B, The length distribution of the *T. guangdongense* proteins identified. C, KOG annotation of the *T. guangdongense* proteins identified.

As shown in Fig. 5A, proteome sequencing yielded a total of 711,861 spectra, of which 28,413 unique spectra were obtained. After removing the peptides with a FDR (false discovery rate) value > 1%, a total of 8430 peptides and 2040 proteins were identified and used to perform the global protein-expression profile analysis across the differential developmental stages. The theoretical molecular weight of the identified proteins varied from 7.76 kDa to 1284.32 kDa, with the majority of them (75.05%) being from 10 kDa to 80 kDa (Fig. 5B and Table S8). Based on the GO analysis (Fig. S4), for BP most of the *T. guangdongense* proteins identified were found to be involved in metabolic process, cellular process, localization and biological regulation. For CC, the majority of proteins were related to the biosynthesis of cell, membrane, and organelle. For MF, numerous proteins were classified into the catalytic and binding activity categories, followed by structural molecule and transporter activity categories. Based on the KOG annotation (Fig. 5C), most proteins were found in the metabolism category including amino acid transport and metabolism, energy production and conversion and lipid transport and metabolism, followed by the cellular processes and signaling categories involved in translational modification, protein turnover, chaperones and signal transduction mechanisms. In addition, 149 proteins were grouped into translation, ribosomal structure and biogenesis. The KEGG pathway analysis showed that most of the proteins were involved in carbohydrate metabolism, amino acid metabolism, translation and protein folding, sorting and degradation pathways (Table S9). It is worth pointing out that a number of proteins that remained unannotated in these databases need further study to explore their function.

### 3.5 Identification and functional annotation of differentially expressed proteins

**Fig. 6:**
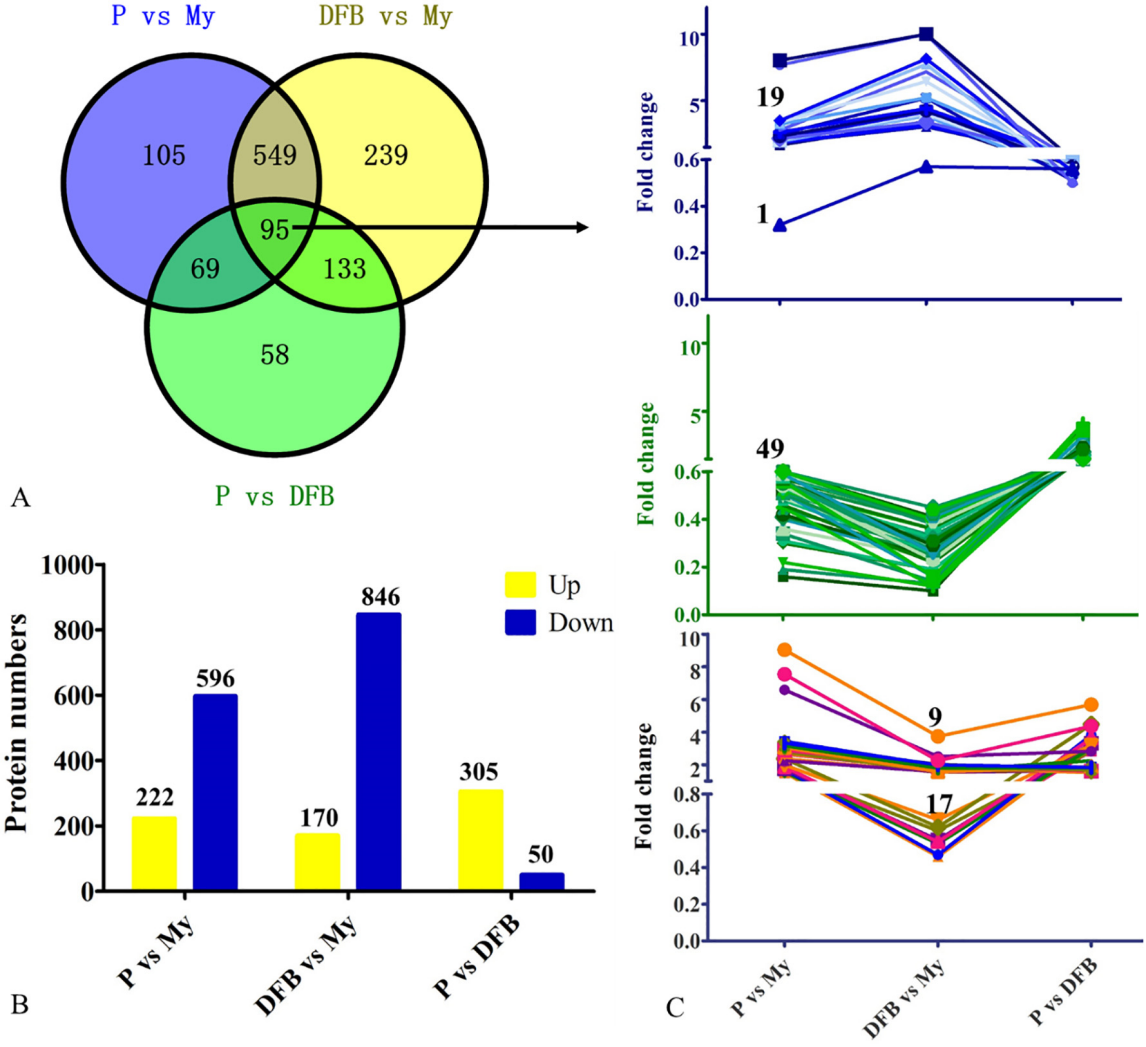
Overview of the number of DEPs number and fold changes in their expression levels in three comparisons of different developmental stages. A, Venn diagram showing the overlap between the total number of DEPs identified in each of the three comparisons. B, Number of upregulation and downregulation proteins for each of the three comparisons. C, Change in the levels of the 95 DEPs shared by each of the three comparisons. My, Mycelial stage; P, Primordial stage; DFB, Developmental fruiting body stage; vs, Versus.

Compared to the My stage, there were 818 and 1016 DEPs identified in comparisons with the P and DFB stages, respectively (Fig. 6A and Table S10). Among these proteins, 222 and 170 proteins were clearly upregulated at the P and DFB stages, respectively, whereas 596 and 846 of these DEPs were significantly downregulated at the P and DFB stages, respectively (Fig. 6B). Comparing the P and DFB stages, 355 proteins were significantly differentially expressed, of which 305 were upregulated and 50 were downregulated. A total of 95 proteins were clearly differentially expressed during the growth and development process of *T. guangdongense* (Fig. 6A). To be more precise, these 95 proteins were clearly differentially expressed in all three comparisons (P vs. My, DFB vs. My and P vs. DFB), suggesting an important role in the process of primordium formation and fruiting body development. As shown in Fig. 6C, 49 of those 95 proteins were clearly downregulated at the P and DFB stages, whereas 19 proteins were significantly upregulated. In addition, 17 of those 95 proteins were upregulated at the P stage but downregulated at the DFB stage, whereas 9 proteins were upregulated at both the P and DFB stages. Only one protein showed an obvious downregulation expression at the P and DFB stages, but was upregulated at he DFB stage compared to the P stage.

An analysis of the GO annotation and KEGG pathways was performed to investigate the function of these DEPs across the differential developmental stages of *T. guangdongense*. The GO annotation results for these DEPs were similar to those of the DEGs in the transcriptome (Table S11). Based on the number of DEPs classified into each functional category, the three largest categories were as follows: cell, cell part and membrane for CC; metabolic process, cellular process and localization for BP; and catalytic activity, binding, and transporter activity for MF. The KEGG pathway analysis revealed that compared to the My stage, the largest number of DEPs in the P and DFB stages were related to the metabolic pathway category, followed by biosynthesis of secondary metabolites in primordia and carbon metabolism (Fig. S5). However, more DEPs were classified into the peroxisome and spliceosome pathways in the comparison of P vs. DFB. Based on the result of the KEGG pathway enrichment analysis, the most significantly enriched pathways (P < 0 0.05) were as follows: four pathways in the comparison of P vs. My stages, including oxidative phosphorylation, meiosis-yeast, mRNA surveillance pathway and synthesis and degradation of ketone bodies; three pathways in the comparison between the P and DFB stages, namely, biosynthesis of unsaturated fatty acids, autophagy-other and alpha-linolenic acid metabolism; and only one pathway (mRNA surveillance) from the comparison of DFB vs. My. In addition, it was found that the majority of DEPs were located to the cytosol, mitochondria, extracellular, nucleus, and plasma membrane (Fig. S6).

### 3.6 Correlation analysis between differentially expressed genes and proteins

**Fig. 7:**
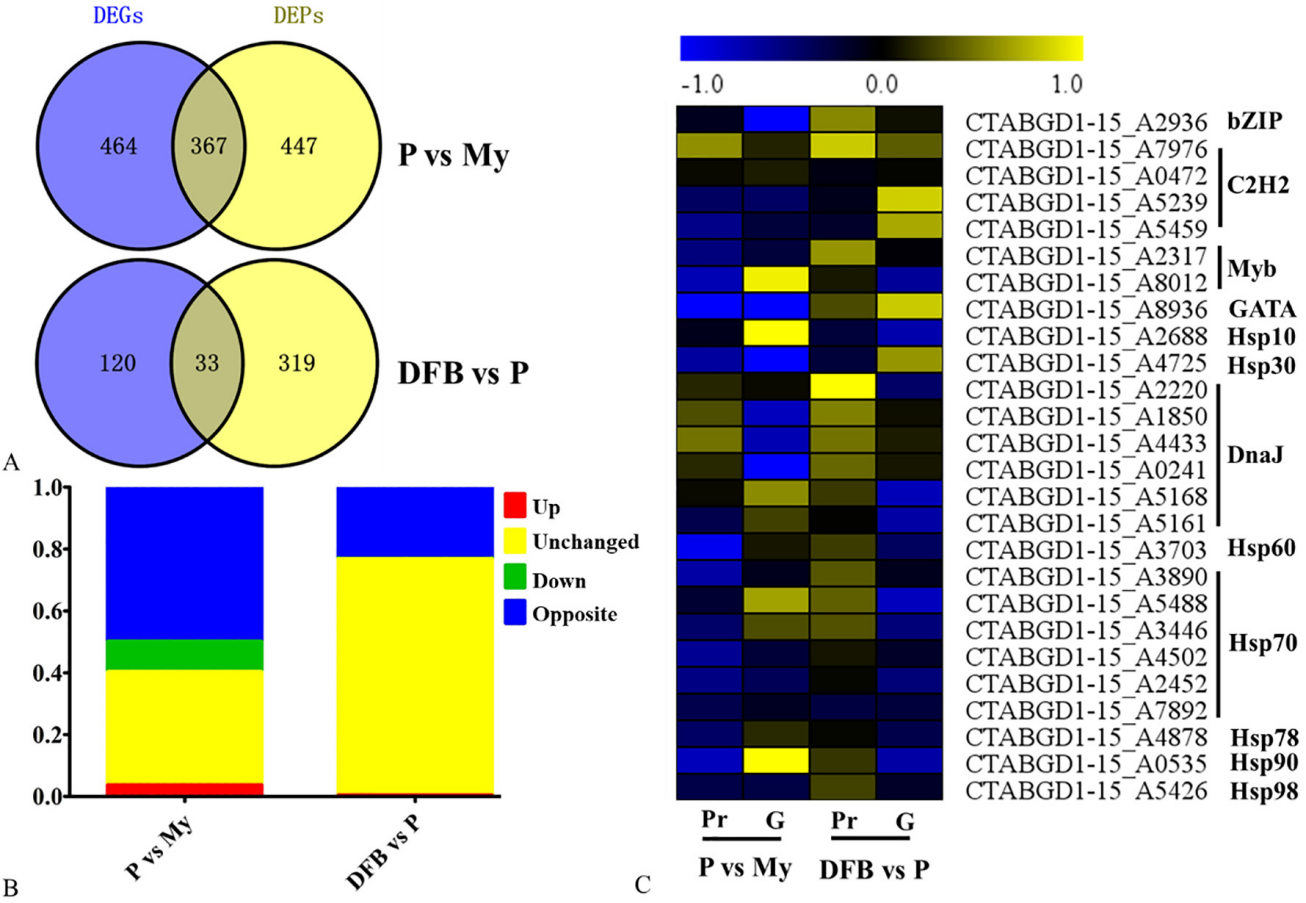
Correlation between the transcriptome and proteome. A, Venn diagram showing the overlap between DEGs and DEPs in the two comparisons. B, Overview of the protein and transcript level changes in two comparisons. C, Heatmap showing the fold changes in heat shock proteins and transcription factors obtained from the correlation analysis. My, Mycelial stage; P, Primordial stage; DFB, Developmental fruiting body stage; vs, Versus; G, Transcript level; Pr, Protein level. Up, Transcript and protein both upregulated. Unchanged, Proteins and transcripts show no changes in expression levels. Down, Transcript and protein both downregulated. Opposite, the opposite changes between transcript and protein levels.

A correlation analysis between the transcriptome and proteome was performed to better understand the molecular mechanisms involved in *T. guangdongense* development. A correlation of the 2022, 2021 and 2004 transcripts and proteins from three comparisons were identified (Table 1). A low degree of correlation between protein level changes and transcript level changes was found for all proteins. Among these, the number of differentially expressed transcripts and proteins correlated were as follows: 367 for P vs. My and 33 for DFB vs. P (Fig. 7A). In the comparison between the P and My stages, the correlated DEGs included 74 upregulated and 198 downregulated genes. Nevertheless, 7 upregulated genes and 1 downregulated gene were found in the genes correlated between the DFB and P stages (Fig. 7B and Table S12). Compared to the differentially expression levels of the transcripts correlated, approximately half of the quantified proteins in the comparison of P vs My showed the opposite changes, whereas proteins with the opposite trend only accounted for 23.20% in the comparison of DFB vs. P (Fig. 7B). Among all the quantified proteins, only one protein (CTABGD1-15_A0198), annotated as an integral membrane protein, showed a similar trend for protein and transcript changes in the P vs. My and DFB vs. P comparisons (Table S12). This difference between mRNA transcript changes and protein changes indicates that changes in mRNA expression levels provide only a limited viewpoint into protein expression changes, and that the developmental process of *T. guangdongense* may be controlled post-transcriptionally through changes in the efficiency of protein translation, protein modifications, and protein interactions.

**Table 1:** Correlation analysis of differentially expressed proteins and genes identified from proteomic and transcriptomic analyses of different developmental stages.

		Gene number	Protein number	Correlation number
P vs. My	All	8952	2040	2022
	DE	3673	818	367
DFB vs. My	All	8963	2039	2021
	DE	3334	1016	365
DFB vs. P	All	8952	2040	2004
	DE	3843	355	33

Note: My, Mycelial stage; P, Primordial stage; DFB, Developmental fruiting body stage; DE, Differential expression.

Heat shock proteins and transcription factors play an important role in regulating the development, metabolism and resistance of an organism to abiotic and biotic stressors. From the correlation analysis between the transcriptome and the proteome, many heat shock proteins and transcription factors were significantly differentially expressed across the developmental stages of *T. guangdongense* (Fig. 7C). Among them, GATA and Hsp30 were clearly downregulated at the P stage at both the transcriptional and translational levels, indicating that they may be involved in primordial germination. Myb (GTABGD1-15_A8012), Hsp60 and Hsp70 (GTABGD1-15_A3890 and GTABGD1-15_A4502) only showed a downregulation of protein levels at the P vs. My stages, but no obvious change found at the mRNA transcriptional level. In contrast, bZIP and DnaJ (GTABGD1-15_A0241) were only downregulated at the mRNA transcriptional level, and Hsp90 showed a clear opposite change at the protein and mRNA transcript levels. In the comparison of DFB vs. P, one DnaJ protein (GTABGD1-15_A2220) was significantly upregulated at the protein level. These results suggest that heat shock proteins and transcription factors have a complex expression pattern and may be involved in growth and development at multiple levels.

### 3.7 Correlation analysis of tryptophan and IAA biosynthesis in *T. Guangdongense*

**Fig. 8:**
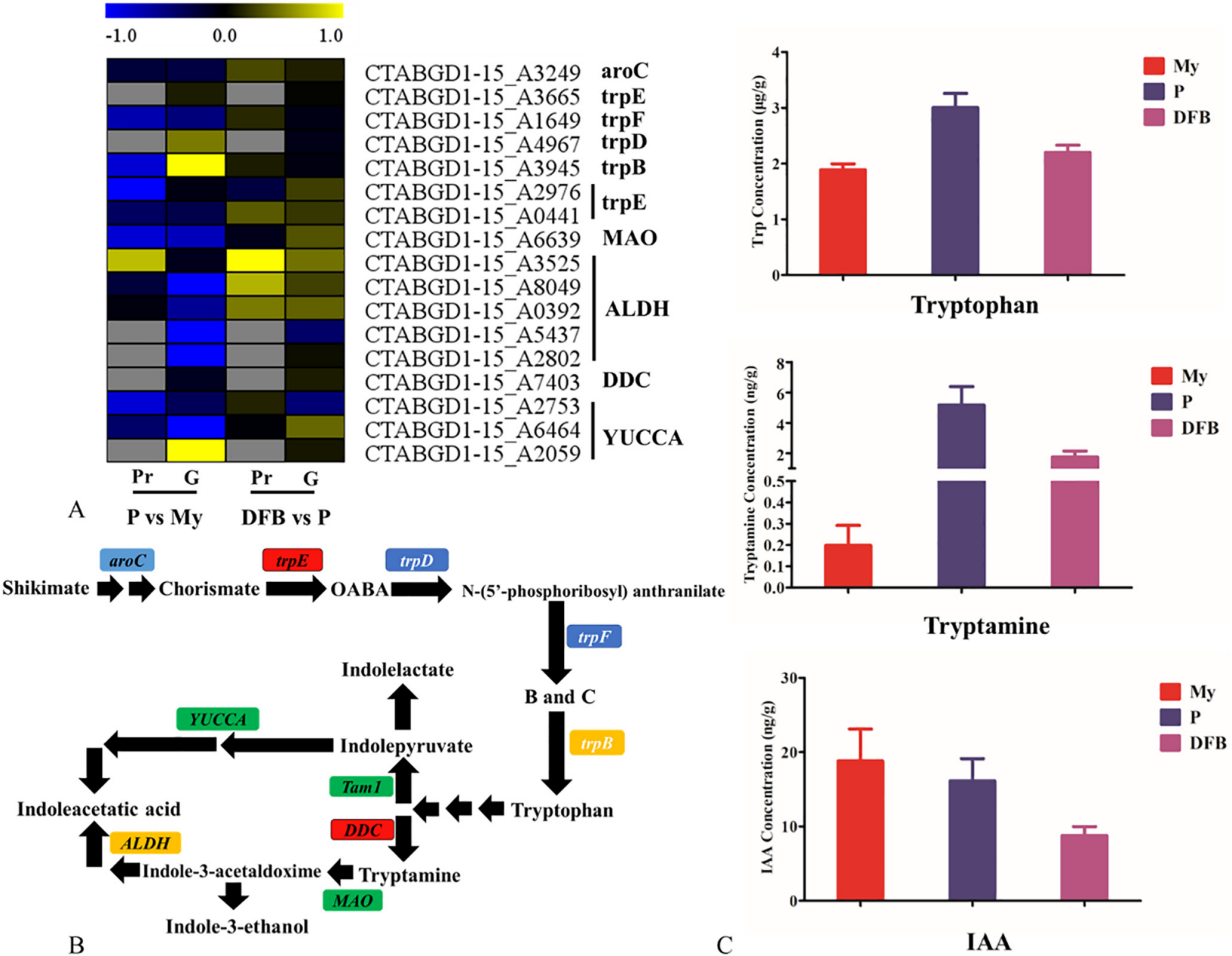
Analysis of tryptophan and IAA biosynthesis on the basis of multi-omics. A, Heatmap showing the expression change of the enzyme proteins related to tryptophan and IAA biosynthesis in the comparisons (P vs. My and DFB vs. P). Gray, Not detected in proteome analysis; Blue, Downregulation; vs, Versus; Yellow, Upregulation; G, Transcript level; Pr, Protein level. B, Overview of the IAA biosynthesis pathway based on the transcriptomic, proteomic and metabolomic data. B and C, Enol- 1-(O-carboxyphenylamino)-1-deoxy ribulose phosphate and Indole-3-glycerol phosphate. C, Contents of tryptophan, tryptamine and IAA in differentially developmental stages of *T. guangdongense*. My, Mycelial stage; P, Primordial stage; DFB, Developmental fruiting body stage. (For interpretation of the references to colour in this figure legend, the reader is referred to the web version of this article.)

Based on an untargeted-metabolomic analysis of mycelia and fruiting bodies of *T. guangdongense* (unpublished data), metabolites related to tryptophan and IAA biosynthesis were identified (Fig. 8B). From the correlation analysis between the transcriptome and the proteome, trpF, Tam-1 (CTABGD1-15_A2976), MAO and YUCCA (CTABGD1-15_A2573) were clearly downregulated at the protein level in the comparison of P vs. My, whereas trpB (tryptophan synthase alpha chain) showed the opposite expression changes at the protein level (downregulation) and the mRNA level (upregulation). In addition, only one aldehyde dehydrogenase protein (CTABGD1-15_A3525) was significantly upregulated in the primordium and fruiting body (Fig. 8A). As shown in Fig. 8C, tryptophan and tryptamine showed a similar trend in changes, and reached a peak at the P stage, indicating a positive effect on the process of primordium formation. Nevertheless, IAA showed a continuous decrease across the developmental process of *T. guangdongense*. These results suggest that IAA can be synthesized via the tryptamine pathway and the indolepyruvate pathway, and that metabolites related to IAA biosynthesis may be involved in the development of *T. guangdongense*.

**Fig. 9:**
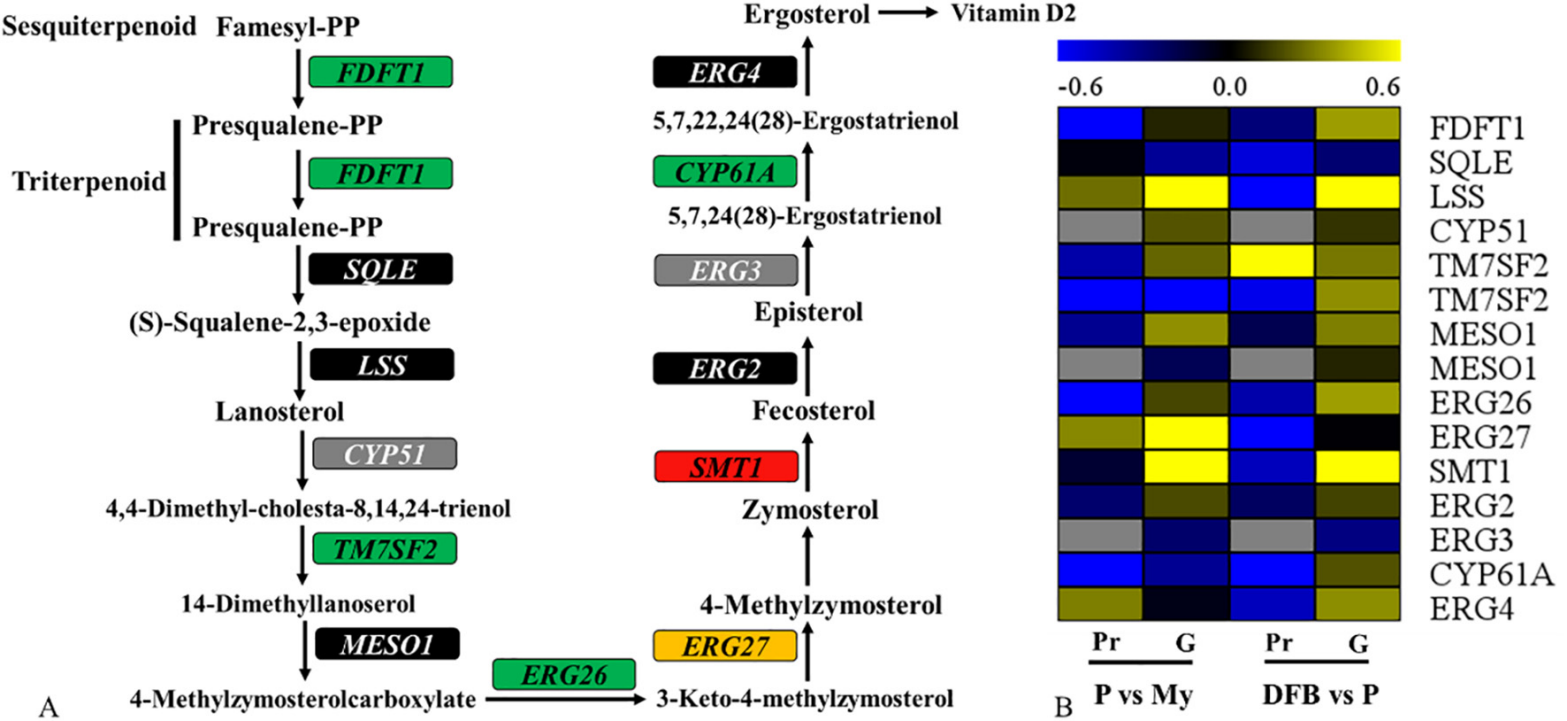
Analysis of terpenoid and ergosterol biosynthesis based on multi-omics. A, Ergosterol biosynthesis pathway based on the transcriptomic and genomic data. B, Heatmap showing the expression change of the enzymes related to terpenoid and ergosterol biosynthesis in the comparisons (P vs. My and DFB vs. P). Gray, Not detected in the proteome analysis; Green, Downregulation at the protein level; Red, Upregulation at the transcript level; Black, No significant change at the protein or transcript levels; Yellow, Complex changes across the different developmental stages; My, Mycelial stage; P, Primordial stage; DFB, Developmental fruiting body stage; G, Transcript level; Pr, Protein level; vs, Versus. (For interpretation of the references to colour in this figure legend, the reader is referred to the web version of this article.)

### 3.8 Correlation analysis of bioactive compounds in *T. Guangdongense*

*T. guangdongense* is able to produce different types of bioactive and nutritional compounds such as polysaccharides, adenosine and terpenoids. These compounds are known to be antioxidants, and possess anti-fatigue, anti-virus, and antitumor activities.

#### 3.8.1 Triterpenoid and ergosterol biosynthesis

A total of 15 key genes involved in terpenoid and ergosterol biosynthesis were identified in the genome and transcriptome of *T. guangdongense*, and 12 enzymes were found in the proteome (Fig. 9A and Table S13). With respect to terpenoid biosynthesis, the protein levels of squalene synthase (FDFT1) were significantly downregulated in the primordia and fruiting bodies compared to the mycelia, but there were no obvious changes found at the transcriptional level (Fig. 9B). With respect to ergosterol biosynthesis, the protein levels of three enzymes, including TM7SF2, ERG26, and CYP61, showed an obvious downregulation in the primordia and fruiting bodies compared to the mycelia, but *TM7SF2* was downregulated in the primordia (Fig. 9B and Table S13). Compared to the protein expression levels in the primordia, TM7SF2 was upregulated in fruiting bodies, whereas LSS and ERG27 were significantly downregulated. Two genes encoding lanosterol synthase (*LSS*) and sterol 24-C-methyltransferase (*SMT1*) were clearly upregulated in the primordia and fruiting bodies compared to the mycelia (Fig. 9B). These results indicate that macro-fungi maybe produce more ergosterol and terpenoids at the My stage than in the FB stages.

#### 3.8.2 Biosynthesis of polysaccharide, adenosine, and other bioactive metabolites

**Fig. 10:**
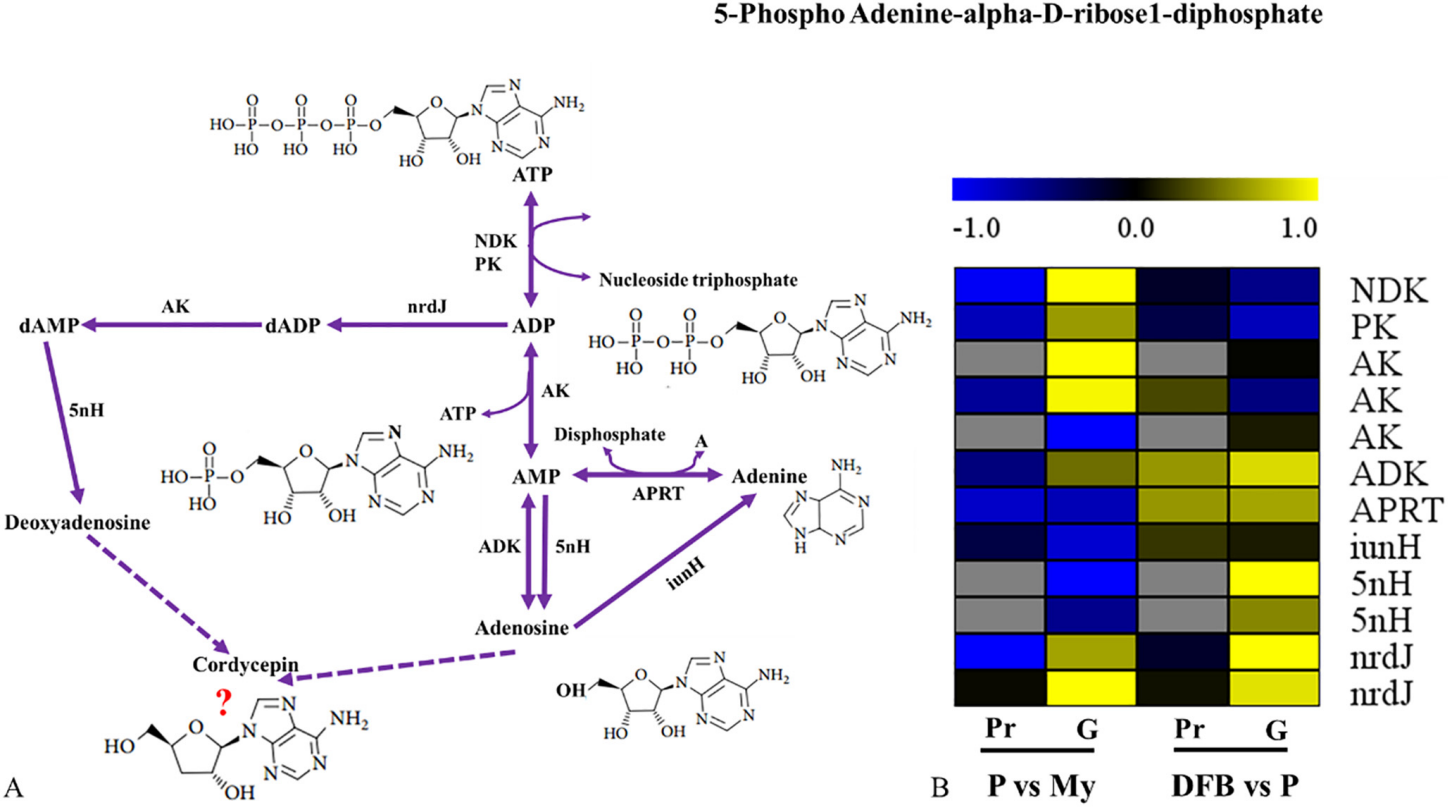
Analysis of adenosine biosynthesis based on multi-omics. A, Adenosine biosynthesis pathway according to the transcriptome and genome data. B, Heatmap showing the expression changes of the enzymes related to adenosine biosynthesis in different comparisons (P vs. My and DFB vs. P). Gray, Not detected in the proteome analysis. My, Mycelial stage; P, Primordial stage; DFB, Developmental fruiting body stage; vs, Versus; G, Transcript level; P, Protein level.

Two genes encoding 1,3-β-glucan synthase and four genes involved in the biosynthesis of 1,6-β-glucans were found on the basis of the functional annotation of the genome (Table S13). Among them, one protein (1,3-β-glucan synthase) showed higher expression level in primordia, but there was an obvious downregulation for UTP-glucose-1-phosphate uridylyltransferase protein, which is important in the biosynthesis of 1,6-β-glucans. With respect to adenosine synthesis (Fig. 10A), the protein levels of five enzymes (NDK, PK, AK, APRT and nrdJ) were all found to be significantly downregulated in primordia, whereas two enzymes (ADK and APRT) were clearly upregulated at the DFB stage (Fig. 10B and Table S13), strongly suggesting that mycelia and fruiting bodies may have higher levels of adenosine. In addition, with regard to those proteins involved the biosynthesis of other bioactive ingredients, nonribosomal peptide synthetase (NRPTS) was upregulated in primordia and fruiting bodies compared to the mycelia, whereas non-ribosomal peptide synthases (NRPSs) was clearly downregulated. Among the eight polyketide synthases (PKSs), half of them exhibited a significant upregulation expression in primordia and fruiting bodies. Further studies need be performed to investigate the production of polysaccharides, adenosine, and other bioactive metabolites at the differential developmental stages of *T. guangdongense*.

### 3.9 Verification of the transcriptome reliability using qRT-PCR

The relative expression levels of 16 genes were used to verify the reliability of the transcriptome data. The expression trends of 14 genes (87.5%) across the differential developmental stages of *T. guangdongense* were consistent with the transcriptome data, with only the absolute fold changes in gene expression showing slight differences (Table S14). Compared to the My stage, DnaJ17 (CTABGD1-15_A4807) at the YFB stage was not differentially expressed by qRT-PCR, but was downregulated in the transcriptome analysis. Compared to the mycelia, Hsp98 (CTABGD1-15_A4807) was differentially expressed in YFB and DFB by qRT-PCR, but was not different in the transcriptome analysis.

## 4 Discussion

Because of its medicinal and nutritional values, *O. sinensis* has historically been regarded as the most valued traditional Chinese medicinal fungus. Recently, numerous transcriptomic, proteomic and metabolomic data have revealed fruiting body and sexual development-related genes [24], the protein uniformity between wild and artificial fruiting bodies [25] and metabolite differences between *O. sinensis* and *C. militaris* [43]. Compared to *O. sinensis*, *T. guangdongense* contains similar metabolites and can be easily artificially cultivated [5]. However, apart from its genome sequence, no omics studies in *T. guangdongense* have been reported. The present work presents the result of a comprehensive analysis of transcriptomic and proteomic data for the differential developmental stages of *T. guangdongense*.

A PCA analysis of the transcriptome data showed that the gene expression patterns in primordia were more similar to fruiting bodies than to the mycelia. In agreement with this, comparisons between the two adjacent stages for primordia and fruiting bodies had fewer differentially expressed genes, confirming the similar gene expression patterns between primordia and fruiting bodies. This is consistent with the gene expression profiles of *O. sinensis* at differential development stages [24], [44]. In this study, a low level correlation was found between the transcriptomic and proteomic data (Fig. 8 B), which could be due to many reasons. First, numerous transcripts or proteins could have very low levels of abundance, which would complicate quantification. In addition, proteins with poor solubility are likely to have a poor recovery rate [45]. The translation efficiency and post-transcriptional regulation such as protein modifications and protein interactions are likely explanations [45], [46]. In addition, sRNAs can affect mRNA stability and promote or inhibit mRNA translation as a means of regulating gene expression, which could result in differences between transcript expression levels and protein expression levels [47], [48], [49]. In the following sections, we discuss the molecular mechanisms involved in fruiting body formation and development, active metabolite biosynthesis and IAA signaling.

### 4.1 Changes in transcription factors during the developmental process.

Transcription factors (TFs) play vital roles in the development and the response of organisms to abiotic and biotic stresses. C2H2 TFs, one type of the common zinc finger proteins, play an important role in regulating ascospore formation, sexual reproduction, and conidial and mycelial growth in *Neurospora crassa* [50], [51]. Knockout of C2H2 TF *Tha00974* decreased biomass and spore production, and reduced its pathogenicity to tomato [52]. In contrast, the fruiting body yield per day of *C2H2* overexpressing strains peaked one day earlier than the wild type *Agaricus bisporus* strain [53]. GATA TFs in fungi have been shown to have various functions and are involved in light response, siderophore biosynthesis, mating-type switching, asexual-sexual development, and appressoria formation [54], [55], [56], [57]. In the present study, a WGCNA analysis showed that 25 TFs including 16 C2H2, 5 bHLH, 2 bZIP, 1 GATA, and 1 HSF were all significantly differentially expressed during the developmental process of *T. guangdongense* (Fig. 4C and Table S6). Based on a correlation analysis between the transcriptomic and proteomic data, 8 TFs including 1 bZIP, 1 GATA, 2 MYB, and 4 C2H2 proteins were found (Table S12). Among them, one C2H2 protein was clearly upregulated at the P stage compared to the My and DFB stages, and one GATA showed a significant downregulation in expression at both the mRNA transcript and protein levels at the P stage compared to the My stage (Fig. 7C), which is consistent with previous studies that *C2H2* showed a higher expression levels in primordia and fruiting [23], [58]. Therefore, we speculate that the P stage is a critical stage for formation of the fruiting body, in which a large number of genes are significantly differentially expressed.

### 4.2 Changes in heat shock proteins during the developmental process

The low correlation between the transcriptome and proteome suggested that multiple protein modifications, including protein folding and degradation, occur in the developmental process of *T. guangdongense*. Heat shock proteins, a stress-responsive family of proteins, are involved in posttranslational modifications, protein folding, aggregation and disaggregation [59]. Based on the WGCNA analysis of the transcriptome data, we found that three *DnaJ* genes and one *Hsp30* gene were significantly downregulated at the P stage compared to the My stage, and that *Hsp30* was differentially expressed in another three comparisons (YFB vs. P, DFB vs. YFB and MFB vs. DFB) (Fig. 4C and Table S7). A total of 18 heat shock proteins were found in the correlation analysis between transcriptome and proteome, and Hsp30 showed an obvious downregulation expression at both the transcript and translation levels at the P stage (Fig. 7C and Table S11). In addition, three Hsp70 proteins and one Hsp90 protein were downregulated at the P stage, and one DnaJ protein showed an obvious downregulation at the DFB stage. It has been reported that the mRNA expression level of heat shock protein 9 in *Grifola frondosa* increased during fruiting body differentiation [60]. In addition, a variety of heat shock proteins such as Hsp25 and DnaJ were involved in mycelial growth, conidial formation and germination, fungal morphogenesis, and the response to environmental factors [61], [62], [63], [64]. The decreased expression of heat shock proteins may be unfavorable for protein and membrane stability during the developmental process of *T. guangdongense*, especially in the presence of detrimental environmental factors.

### 4.3 Changes in the MAPK pathway during the developmental process

Mitogen-activated protein kinase (MAPK) pathways, being composed of three serine/threonine protein kinases (MAPKKK, MAPKK and MAPK), can transduce extracellular signals to regulate transcriptional events and other cellular processes [65]. They have been reported to be involved in the formation of the ascocarp in ascomycetes, pathogenicity in insects, sexual reproduction, osmolarity resistance, and cell wall integrity in fungi [66], [67], [68], [69]. A total of 12 genes encoding serine/threonine protein kinases were identified using a correlation between the transcriptomic and proteomic data (Table S12). Among them, four MAPK proteins showed obvious differential expression at the P stage compared to the My and DFB stages, indicating there is a relationship between primordium formation and environmental factors such as light and temperature. In addition, the differential expression of MAPKs can result in altered regulation of TFs and other related proteins, and thus may be involved in the developmental process of *T. guangdongense*.

### 4.4 Changes in proteins related to IAA and bioactive metabolites during development process

As the first discovered plant hormone, IAA was also found in basidiomycete and ascomycete fungi [70], [71]. In plants, a low concentration of IAA effectively promotes the growth of roots and leaves and resistance to salt, low temperatures, and heavy metal stresses [72], [73], [74]. The current study showed that many genes related to IAA biosynthesis were obviously downregulated at the P and DFB stages, and that the IAA content showed a continuous decrease with the development of fruiting bodies (Fig. 8). In the basidiomycete fungus *L. edodes*, exogenous auxin and its analogues can alleviate the effects of oxidative damage induced by heat stress [75]. Therefore, we speculate that IAA may regulate the development and the response of *T. guangdongense* toward abiotic stresses, but this needs to be further confirmed.

The main bioactive ingredients identified in *T. guangdongense* are adenosine, polysaccharides, terpenoids, etc. [10], [11]. In the adenosine biosynthesis pathway of *O. sinensis*, most of the key genes involved in this pathway were significantly downregulated at the late stage of fermentation [76]. Similarly, our results suggest that many proteins involved in adenosine biosynthesis are significantly downregulated at the P and DFB stages compared to the My stage (Fig. 10B and Table S13). Of all the medicinal fungus polysaccharides, the water-soluble 1,3-β- glucans and 1,6-β-glucans show the best immunomodulatory and antioxidant effects [77], [78]. Three genes involved in 1,3-β- glucan and 1,6-β-glucan biosynthesis showed clearly differential expression at the P stage compared to My or DFB stages (Table S13). Seven of 15 key proteins involved in the biosynthesis of terpenoids and ergosterol were also differentially expressed across the developmental process of *T. guangodngense*. These results indicate that *T. guangdongense* may be able to produce more ergosterol, terpenoids, and adenosine at the My stage than at the P and DFB stages.

Cordycepin is an important metabolite in *C. militaris*, and possesses the potential as a medicine, food and herbicide [79], [80], [81]. In *T. guangdongense*, extremely low levels of cordycepin were detected, and the genes involved in cordycepin biosynthesis were not identified in the genome of *T. guangdongense*. Nevertheless, it contained high levels of cordycepic acid, adenosine, ergosterol, polysaccharides, and vitamins, suggesting the potential of *T. guangdongense* as a food and a medicine, although this need further research. In future studies, we will comprehensively analyze cordycepin using multiple approaches, and explore the biosynthesis mechanisms of metabolites using reverse genetics methods. In addition, we will conduct in vivo studies using extracts of *T. guangdongense* to investigate the functions of *T. guangdongense* bioactive metabolites.

## 5 Conclusions

In this study, a combination of transcriptomic and proteomic analyses were used to provide a view of hub genes, key pathways, development-related and metabolite-related gene/protein profiles during the differential developmental stages of *T. guangdongense*. It was found that the transcriptomic profile at the My stage was significantly different from the P and FB stages. Many functional genes and proteins, especially associated with transcriptional regulation, protein modification, signal transduction and the biosynthesis of bioactive metabolites, were identified. Most of the differentially expressed TFs belonged to the C2H2 family, and numerous heat shock proteins such as Hsp30 and DnaJ showed significant differential expression during the developmental process. Moreover, IAA levels and key enzymes involved in its biosynthesis were downregulated, suggesting a potential effect on the growth and development of *T. guangdongense*. Furthermore, numerous proteins related to the biosynthesis of adenosine, terpenoid, ergosterol, and polysaccharide were differentially downregulated at the P and FB stages. In summary, the present study sheds lights on the developmental process of *T. guangdongense* and provides information on the biosynthesis of several metabolites. It is hoped that these data will lead to a further dissection of the functions of the DEGs identified here, and this will benefit the development of both industrial and medical applications of this medicinal fungus.

## CRediT authorship contribution statement

**Gangzheng Wang:** Conceptualization, Methodology, Data curation, Validation, Writing - original draft. **Min Li:** Resources, Validation, Data curation. **Chenghua Zhang:** Conceptualization, Methodology. **Huijiao Cheng:** Resources, Investigation. **Yu Gao:** Resources, Investigation. **Wangqiu Deng:** Conceptualization, Writing - review & editing. **Taihui Li:** Conceptualization, Writing - review & editing.

## Declaration of Competing Interest

The authors declare that they have no known competing financial interests or personal relationships that could have appeared to influence the work reported in this paper.
