# Supplementary material for: Transcriptome and proteome analyses reveal the regulatory networks and metabolite biosynthesis pathways during the development of Tolypocladium guangdongense
Source: Comput Struct Biotechnol J. 2020 Jul 25;18:2081–94. doi: 10.1016/j.csbj.2020.07.014 (PMC7419252; doi:10.1016/j.csbj.2020.07.014)

**Figure S1** The correlation analysis among all the replicates.

**
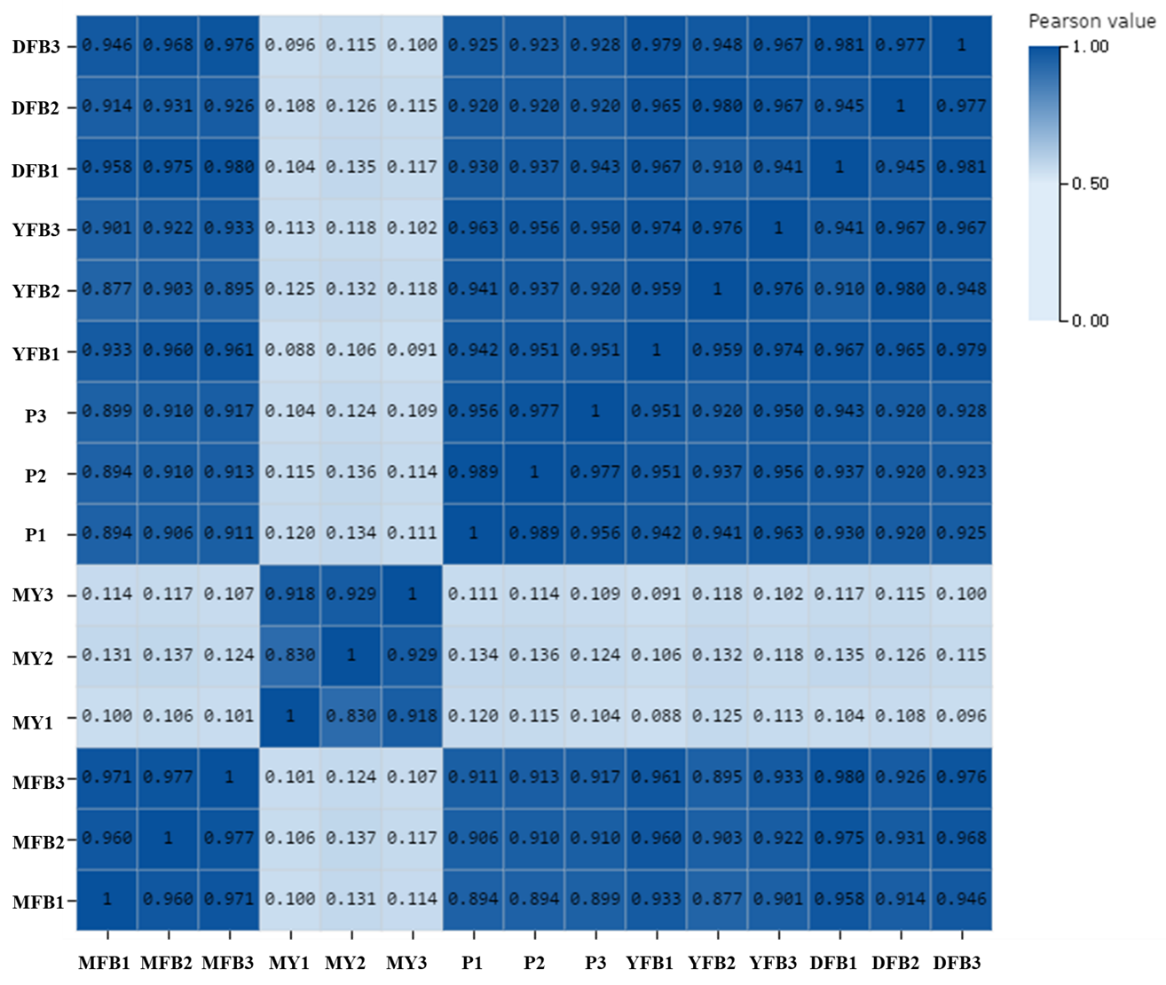
**

**Figure S2** Principal component analysis of the RNA-Seq data.

**
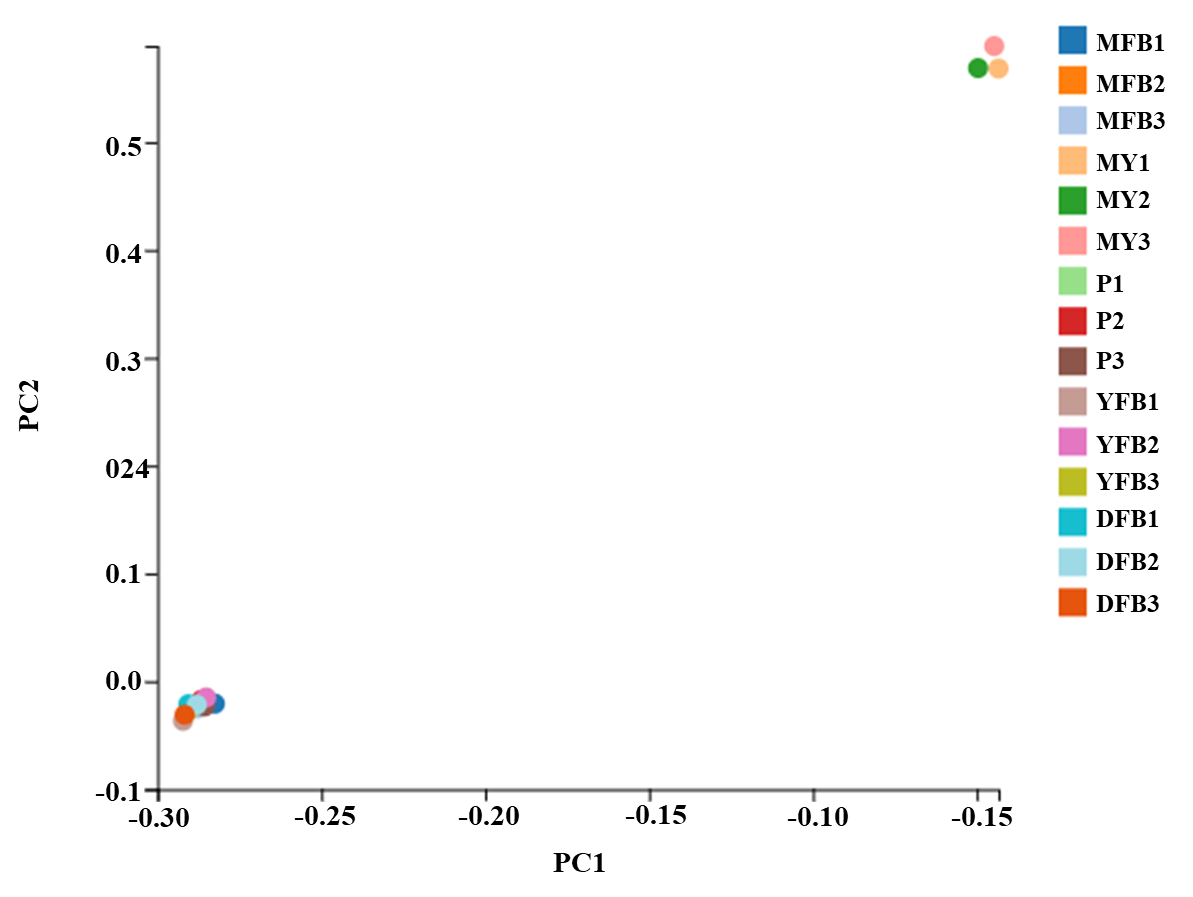
**

**Figure S3** KEGG pathway enrichment analysis of hub genes identified by WGCNA in two adjacent stages.

**
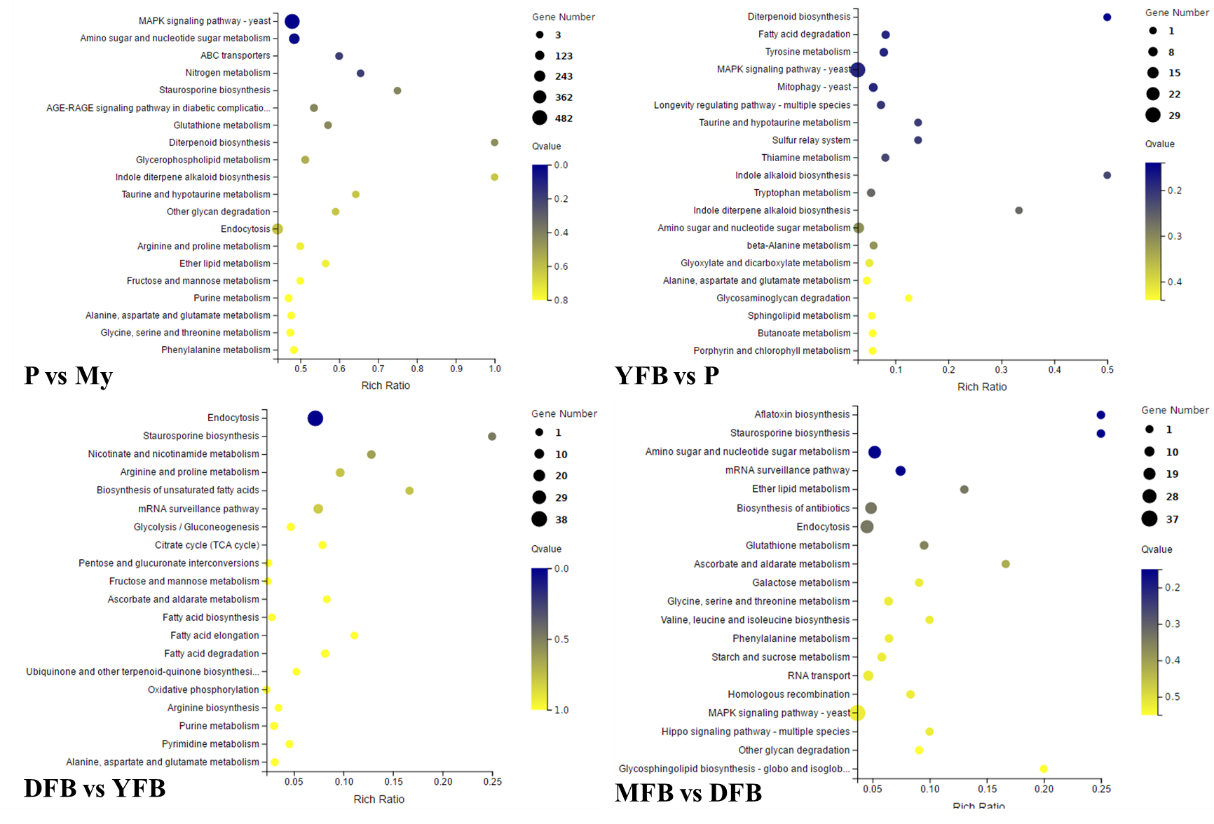
**

**Figure S4** GO annotation of the identified *C. guangdongensis* proteins.


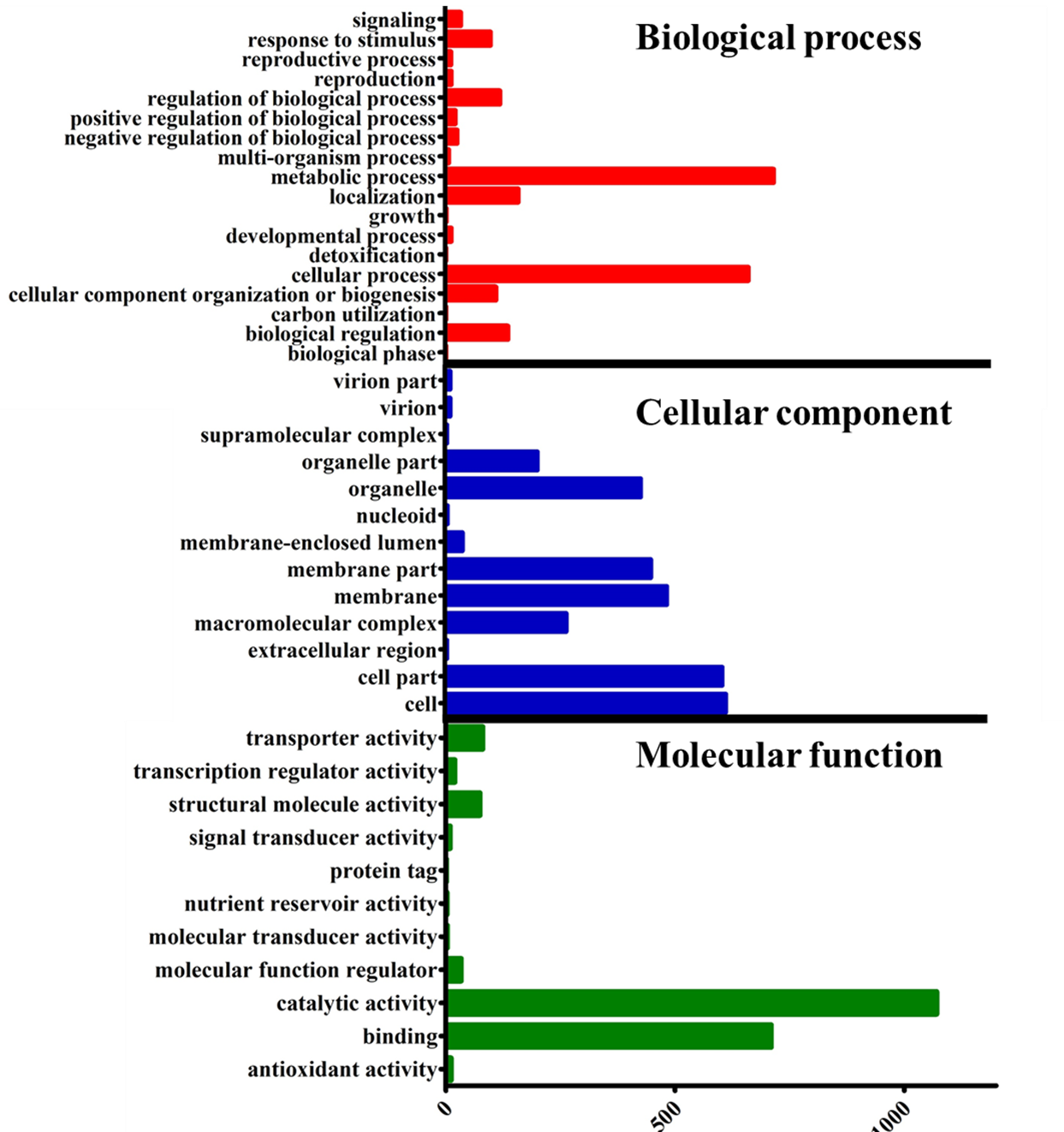


**Figure S5** KEGG pathway analysis of DEPs in three developmental stages. A, The enrichment KEGG pathway analysis of DEPs in the comparison between P and My stages. B, The enrichment KEGG pathway analysis of DEPs in the comparison between DFB and My stages. C, The enrichment KEGG pathway analysis of DEPs in the comparison between P and DFB stages. My, Mycelial stage; P, Primordial stage; DFB, Developmental fruiting body stage.


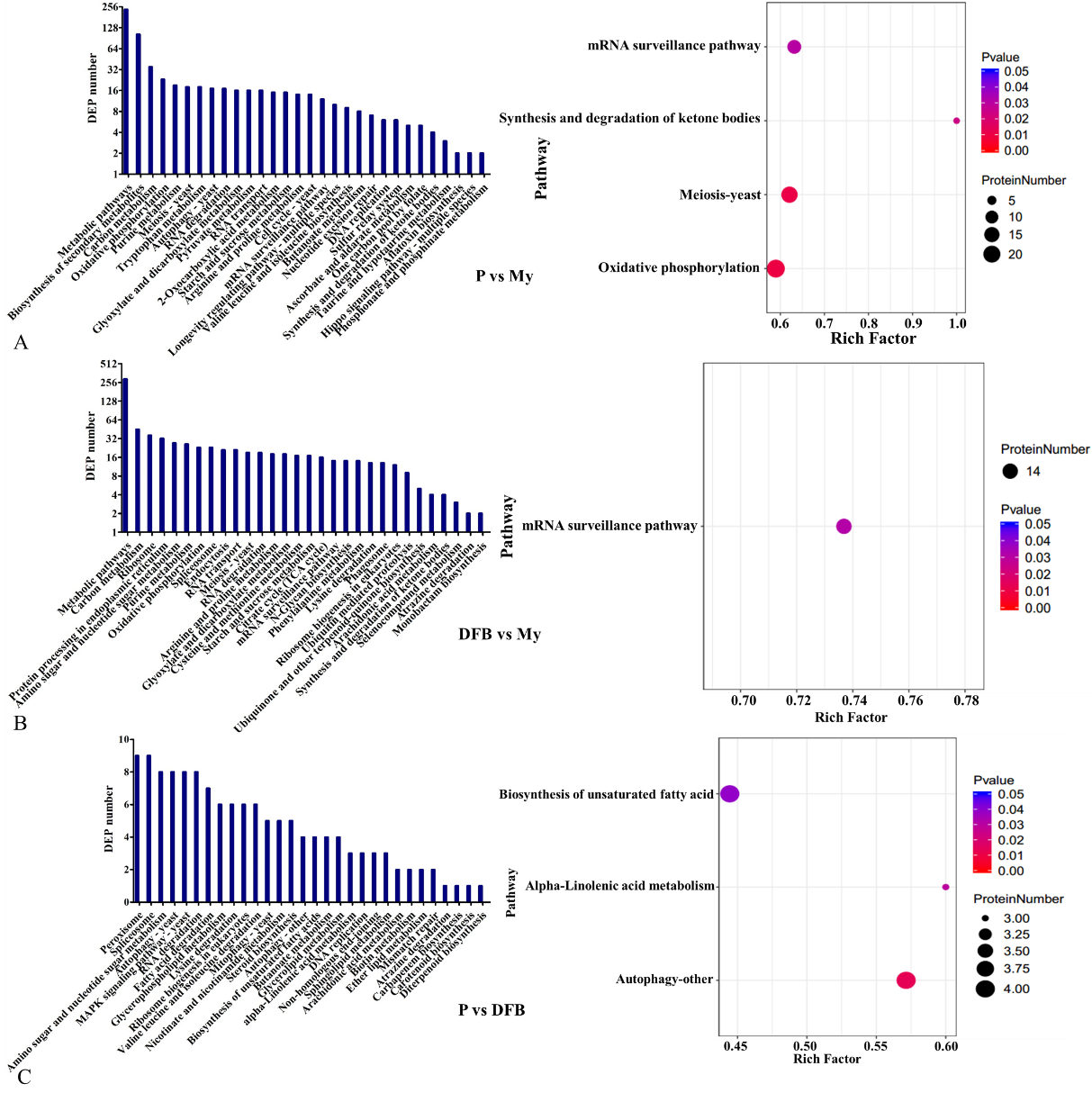


**Figure S6** Bar plot showing DEP number on the basis of subcellular localization prediction. My, Mycelial stage; P, Primordial stage; DFB, Developmental fruiting body stage.


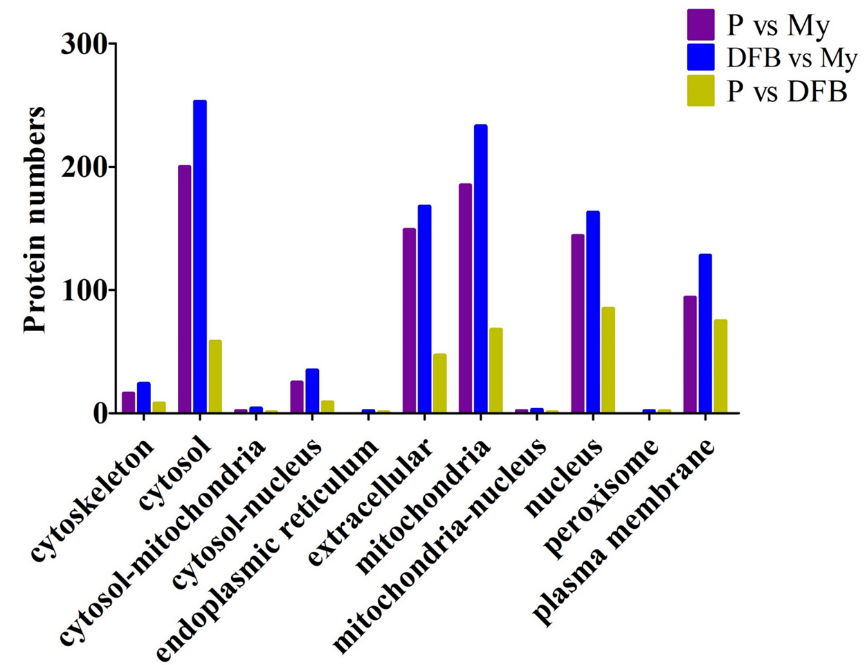

Supplement: Supplementary data 2 [file mmc2.docx]
